# α-Radioimmunotherapy with ^213^Bi-anti-CD38 immunoconjugates is effective in a mouse model of human multiple myeloma

**DOI:** 10.18632/oncotarget.2986

**Published:** 2014-12-10

**Authors:** Katharina Teiluf, Christof Seidl, Birgit Blechert, Florian C. Gaertner, Klaus-Peter Gilbertz, Vanesa Fernandez, Florian Bassermann, Jan Endell, Rainer Boxhammer, Stephane Leclair, Mario Vallon, Michaela Aichler, Annette Feuchtinger, Frank Bruchertseifer, Alfred Morgenstern, Markus Essler

**Affiliations:** ^1^ Department of Nuclear Medicine, Technische Universität München, Munich, Germany; ^2^ Department of Obstetrics and Gynecology, Technische Universität München, Munich, Germany; ^3^ Department of Nuclear Medicine, Universitätsklinikum Bonn, Bonn, Germany; ^4^ Institute of Radiobiology, German Armed Forces, Munich, Germany; ^5^ III. Medical Department, Technische Universität München, Munich, Germany; ^6^ MorphoSys AG, Martinsried/Planegg, Germany; ^7^ Division of Hematology, Stanford University School of Medicine, Stanford, CA, USA; ^8^ Institute of Pathology, Helmholtz Zentrum München, Neuherberg, Germany; ^9^ European Commission, Joint Research Centre, Institute for Transuranium Elements, Karlsruhe, Germany

**Keywords:** anti-CD38-MAb, α-emitter ^213^Bi, OPM2 cells, radioimmunotherapy, cell death

## Abstract

In spite of development of molecular therapeutics, multiple myeloma (MM) is fatal in most cases. CD38 is a promising target for selective treatment of MM. We tested radioimmunoconjugates consisting of the α-emitter ^213^Bi coupled to an anti-CD38 MAb in preclinical treatment of MM. Efficacy of ^213^Bi-anti-CD38-MAb was assayed towards different MM cell lines with regard to induction of DNA double-strand breaks, induction of apoptosis and initiation of cell cycle arrest. Moreover, mice bearing luciferase-expressing MM xenografts were treated with ^213^Bi-anti-CD38-MAb. Therapeutic efficacy was monitored by bioluminescence imaging, overall survival and histology. ^213^Bi-anti-CD38-MAb treatment induced DNA damage which did not result in activation of the G2 DNA-damage-response checkpoint, but instead in mitotic arrest and subsequent mitotic catastrophe. The anti-tumor effect of ^213^Bi-anti-CD38-MAb correlated with the expression level of CD38 in each MM cell line. In myeloma xenografts, treatment with ^213^Bi-anti-CD38-MAb suppressed tumor growth via induction of apoptosis in tumor tissue and significantly prolonged survival compared to controls. The major organ systems did not show any signs of ^213^Bi-induced toxicity. Preclinical treatment of MM with ^213^Bi-anti-CD38-MAb turned out as an effective therapeutic option.

## INTRODUCTION

Multiple myeloma (MM) is a hematological malignancy characterized by infiltration of the bone marrow by malignant plasma cells. It accounts for 13% of hematological disorders [1] and for roughly 20% of deaths from hematological cancer worldwide [2]. Patients suffering from MM are treated with high-dose chemotherapy in combination with autologous stem cell transplantation [3, 4]. Additional application of new therapeutic agents such as the proteasome inhibitor bortezomib and the immunomodulatory drugs thalidomide and lenalidomide has further increased progression-free survival and contributed to improvement in overall survival [4].

However, despite these proceedings, MM remains an incurable disease as most patients relapse due to multidrug resistance. Therefore new therapeutic options are urgently needed. These include the new proteasome inhibitors carfilzomib, marizomib and MLN 0897 [5] as well as the immunomodulatory drug pomalidomide, inhibitors of the phosphatidylinositol 3-kinase pathway [6] and histone deacetylase inhibitors [7]. Also compounds targeting cytokines or accessory cells in the bone-marrow microenvironment have shown promising results [8]. Another successful approach is the application of therapeutic monoclonal antibodies specifically targeting MM [9]. Several new therapeutic MAbs have been developed targeting the antigens β_2_M, IL-6, HM1.24, CD70, CD74, CD40, CS1, and CD38, all associated with MM [9]. The anti-CS1 MAb elotuzumab triggered tumor regression in preclinical models of MM and showed promising response rates in patients with relapsed or refractory MM after combined administration of elotuzumab, lenalidomide and dexamethasone [10, 11].

CD38 is another promising target used for immunotherapy because MM cells mostly show overexpression of CD38 [12]. The anti-CD38 antibody daratumumab eradicates plasma cells derived from MM patients and has demonstrated therapeutic efficacy in a preclinical model. The therapeutic potential of daratumumab is currently investigated in a phase I/II study [13]. Two more anti-CD38 MAbs, SAR650984 and MOR03087, are presently being analysed in clinical trials, highlighting the potential significance of CD38 as a therapeutic target in MM [14, 15].

Coupling of radionuclides may potentiate direct anti-tumor effects of monoclonal antibodies such as of CD38. Especially α-particle emitting isotopes may be valuable in eradication of tumor cells because α-particles show a comparatively short range in tissue (40 to 100 μM) combined with a high energy (4 - 9 MeV). Therefore, the linear energy transfer (LET), i.e. the linear rate of loss of energy by an ionizing particle traversing soft tissue, of α-emitters is comparatively high (50 – 230 keV/μm).

The efficacy of targeted therapy with α-emitters has been demonstrated in an increasing number of preclinical and clinical studies [16-18]. Therefore, the aim of our study was to assay the efficacy of radioimmunoconjugates composed of the α-emitter ^213^Bi coupled to the anti-CD38 MAb MOR03087 in targeted treatment of MM. Anti-myeloma activity of ^213^Bi-anti-CD38-MAb was investigated *in vitro* in terms of induction of DNA double-strand breaks, initiation of cell-cycle arrest in the G2/M-phase and eradication of MM cells as well as in a preclinical model of MM investigating tumor development, intratumoral apoptosis and survival of animals.

## RESULTS

### Binding of anti-CD38-MAb and CHX-A”-DTPA chelated anti-CD38-MAb to OPM2 cells

**Figure 1:**
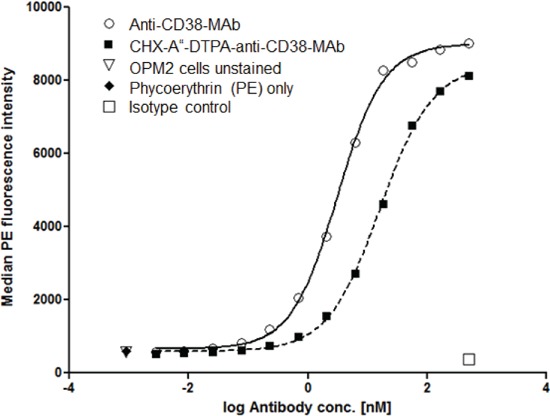
Binding affinity of native and chelated anti-CD38-MAb Binding of the native anti-CD38 monoclonal antibody MOR03087 before and after coupling of the chelating agent CHX-A”-DTPA to OPM2 cells was assayed by flow cytometry. EC_50_ values were 3.1 and 16.4 nM, respectively.

Anti-CD38-Mab was coupled to CHX-A”-DTPA as described in the Methods section. To determine the binding affinity, we measured EC_50_ values for coupled and native antibodies. As shown in Fig. 1, EC_50_ of anti-CD38-Mab was 3.1 nM whereas the EC_50_ of CHX-A”-DTPA-anti-CD38-MAb was 16.4 nM, indicating that the affinity of the conjugate is lower compared to the native antibody, but still appropriate for therapy. These results correspond to 29,951.5 ± 937.0 molecules of anti-CD38 MAb bound per OPM2 cell.

### Correlation of ^213^Bi-anti-CD38-MAb binding to myeloma cell lines and cytotoxicity

**Figure 2:**
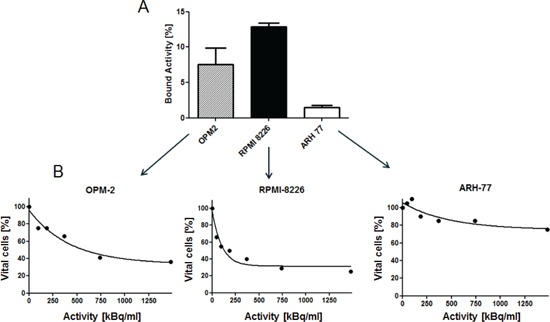
Correlation of Bi-anti-CD38-MAb binding and cytotoxicity A) Percentages of ^213^Bi-anti-CD38-MAb binding to the multiple myeloma cell lines OPM2, RPMI8226 and ARH77 as quantified by bound ^213^Bi activity in the cell pellet. B) Assessment of cytotoxicity of ^213^Bi-anti-CD38-MAb upon OPM2, RPMI and ARH77 myeloma cells as quantified by the CellTiter96® cell proliferation assay 48 h after initiation of treatment.

Binding of ^213^Bi-anti-CD38-MAb to the myeloma cell lines RPMI8226, OPM2, and ARH77 was different. The percentage of bound ^213^Bi-labelled antibody was 13.0% in RPMI cells, 7.5% in OPM2 cells and 1.2% in ARH77 cells (Fig. 2A) indicating different CD38-expression in the investigated cell lines. Accordingly, the anti-tumor effect of ^213^Bi-anti-CD38-MAb was different in each cell line. LD_50_ values for ^213^Bi-anti-CD38-MAb activity concentrations amounted to 0.185 MBq/ml, 0.555 MBq/ml, and > 1.85 MBq/ml for RPMI, OPM2 and ARH cells, respectively, as determined by CellTiter96® cell viability assay (Fig. 2B).

### ^213^Bi-anti-CD38-MAb induced DNA double-strand breaks in OPM2 and ARH77 cells

**Figure 3:**
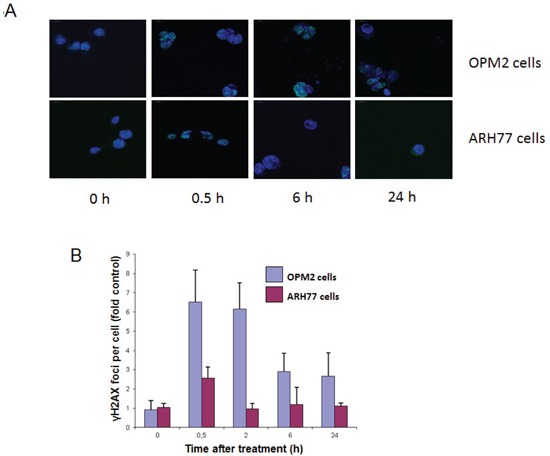
Quantification of ^213^Bi-anti-CD38-MAb induced DNA double strand breakes OPM2 or ARH77 multiple myeloma cells were treated with ^213^Bi-anti-CD38-MAb (1.48 MBq/ml) for 3 h at 4°C to prevent DNA-repair. Subsequently cells were washed with PBS and incubated at 37°C in fresh medium. At the indicated time points cells were stained for γH2AX (A) and the signals (foci per cell) were quantified using Definiens© software (B).

Induction of DNA double-strand breaks by treatment with ^213^Bi-anti-CD38-MAb (1.48 MBq/ml for 3 h at 4°C) was different in OPM2 and ARH77 cells according to the different cell binding of ^213^Bi-anti-CD38 immunoconjugates (Fig. 3A). At 0.5 h after treatment numbers of γH2AX foci per cell reached a maximum for both cell lines, however in OPM2 cells number of γH2AX foci was approximately 2.5 fold higher compared to ARH77 cells. In OPM2 cells number of γH2AX foci decreased with time but did not reach control values even after 24 h. In contrast, in ARH77 cells control values were already reached 2 h after incubation with ^213^Bi-anti-CD38-MAb (Fig. 3B). This could be due to the comparatively low number of induced γH2AX foci or to a better repair capacity of ARH77 cells compared to OPM2 cells.

### ^213^Bi-anti-CD38-MAb induces mitotic cell-cycle arrest and subsequent mitotic catastrophe in OPM2 cells

**Figure 4:**
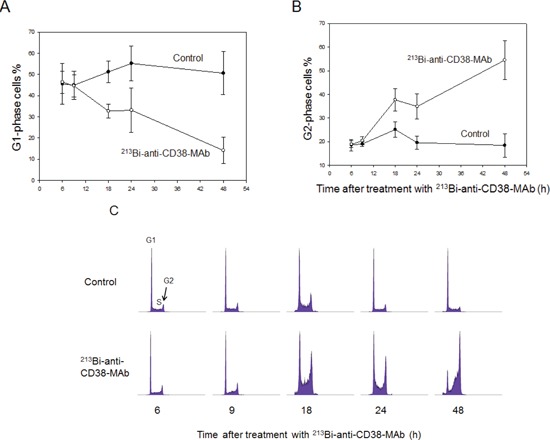
^213^Bi-anti-CD38-MAb induced cell cycle arrest Percentages of OPM2 cells in the G1-phase (A) and the G2-phase (B) of the cell cycle were determined at the indicated time points after treatment with ^213^Bi-anti-CD38 immunoconjugates (1.48 MBq/ml, 3 h, 37°C). Means of three independent experiments ± SD are shown. In C) the corresponding histograms of one representative experiment are depicted.

**Figure 5:**
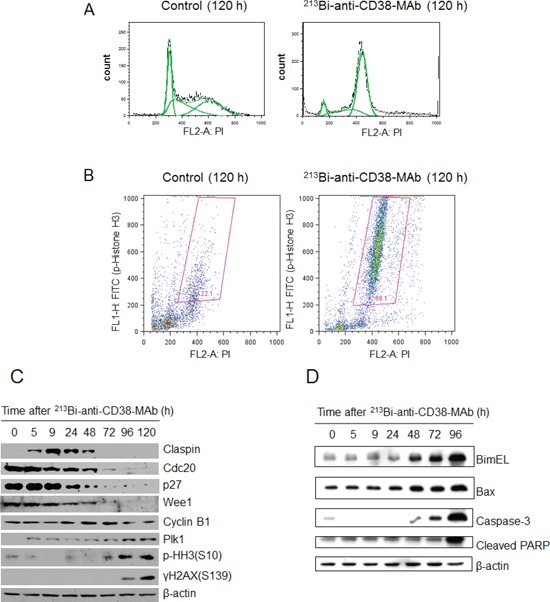
Signal mechanisms triggering ^213^Bi-anti-CD38-MAb induced cell cycle arrest and apoptosis A) DNA content and B) histone H3 phosphorylation in OPM2 cells 120 h after treatment with ^213^Bi-anti-CD38-MAb (0.74 MBq/ml) or PBS (control) as determined by flow cytometry. C) Expression of major G2/M checkpoint activating and pro-apoptotic proteins as well as D) caspase-3 activation and PARP cleavage at different time points after incubation of OPM2 cells with ^213^Bi-anti-CD38-MAb (0.74 MBq/ml) as determined by immunoblotting.

Cell cycle arrest of OPM2 cells following treatment with ^213^Bi-anti-CD38-MAb (1.85 MBq/ml) for 3 h at 37°C) was investigated by flow cytometry. The percentage of OPM2 cells arrested in G2 phase increased at 12 h, 18 h and 24 h after treatment and reached a maximum of 55% at 48 h. Concurrently the percentage of OPM2 cells in G1 phase dropped below 15% at 48 h. In contrast, the level of untreated OPM2 cells (controls) in G2 and G1 phase remained constant at approximately 20% and 50%, respectively, throughout the observation period (Fig. 4A/B). The results are illustrated using representative histograms showing the proportions of cells in G1, S and G2 phase in untreated and ^213^Bi-anti-CD38-MAb treated OPM2 cells (Fig. 4C). To further characterize the cell cycle phase in which the cells are arrested, dual parameter flow cytometry with phospho-histone H3 staining was performed. Histone H3 is phosphorylated at serine 10 upon entrance of cells into mitosis and phosphorylation correlates with mitotic chromosome condensation [19]. As shown in Fig. 5A, 120 h after treatment with ^213^Bi-anti-CD38-MAb (0.74 MBq/ml), OPM2 cells were arrested with a 4n DNA content, indicative of a G2/M arrest, and as shown in Fig. 5B, demonstrate a strong increase in histone H3 phosphorylation, indicating that cells had entered mitosis despite the treatment with DNA damaging α-irradiation [19]. To further elucidate the molecular mechanisms by which cells treated with ^213^Bi-anti-CD38-MAb are able to bypass the G2/M checkpoint to enter mitosis, cell extracts were also analyzed by immunoblotting. Major G2/M checkpoint activating events like claspin and Wee1 stabilisation, as well as Plk1 destabilization [20] were absent despite the presence of DNA damage, as evidenced by the phosphorylation of histone H2AX (γH2AX) (Fig. 5C). Instead, cells entered mitosis (shown by phosphorylation of histone H3) and underwent subsequent apoptosis as demonstrated by the cleavage of PARP and pro-caspase 3 (Fig. 5D). Of notice, substantial stabilization of pro-apoptotic BimEL (Bcl-2 interacting mediator of cell death, extra long form), was observed (Fig. 5D) supporting the notion that BimEL may be involved in this mitotic cell death event. Based on these observations, it seems obvious that ^213^Bi-anti-CD38-MAb treatment of OPM2 cells induces significant DNA damage, which however does not result in the activation of the G2 DNA-damage-response checkpoint, but instead in mitotic arrest and subsequent mitotic catastrophe.

### Preclinical treatment of MM with ^213^Bi-anti-CD38-MAb

**Figure 6:**
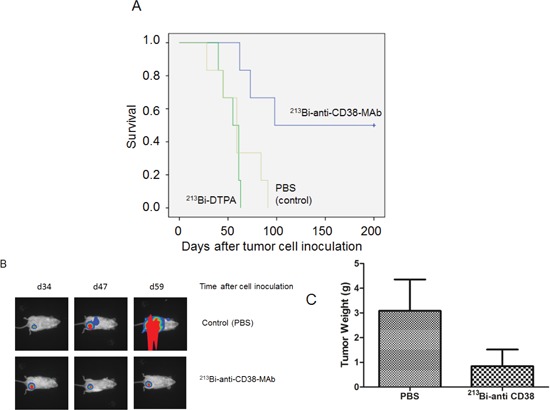
Preclinical treatment study A) Kaplan-Meyer plot showing the survival of mice after six treatments with PBS (control), unspecific ^213^Bi-DTPA, and ^213^Bi-anti-CD38-MAb (1.85 MBq, respectively) between days 25 and 42 after OPM2 tumor cell inoculation. B) Bioluminescence imaging of tumor development at days 34, 47, and 59 after cell inoculation in PBS treated control mice and in animals treated with ^213^Bi-anti-CD38-MAb. C) Weights of tumor tissue two weeks after the final treatment cycle in mice treated with PBS (control) and ^213^Bi-anti-CD38-MAb.

In mice bearing OPM2 xenografts repeated treatment with ^213^Bi-anti-CD38-MAb prolonged survival compared to treatment with unspecific ^213^Bi-DTPA or PBS. Median survival was 60 days in the PBS group, 55 days in the ^213^Bi-DTPA group and 100 days in the ^213^Bi-anti-CD38 group. Three animals of the group treated with ^213^Bi-anti-CD38-MAb survived longer than 200 days (Fig. 6A). As demonstrated by non-invasive bioluminescence imaging in two animals, tumor size remained constant after treatment with ^213^Bi-anti-CD38-MAb at days 34, 47, and 59 after tumor cell inoculation, whereas it drastically increased in PBS treated mice (Fig. 6B). Accordingly, the tumor weights were significantly different in ^213^Bi-anti-CD38-MAb and PBS treated mice (Fig. 6C).

### ^213^Bi-anti-CD38-MAb induced cell death in xenograft tumor tissue

**Figure 7:**
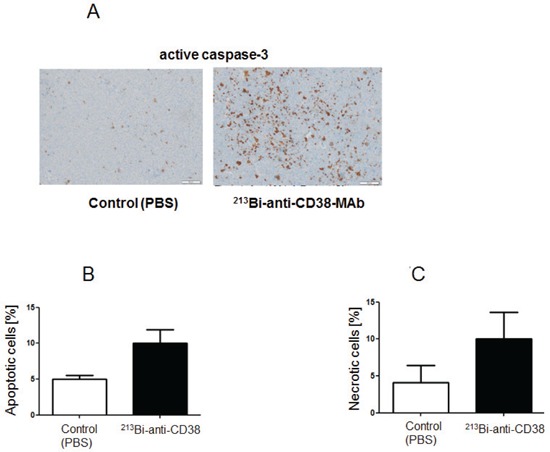
Assessment of intratumoral apoptosis and necrosis Two weeks after therapy intratumoral apoptosis and necrosis were determined on paraffin slices of tumor tissue. A) Immunohistochemical detection of apoptotic cells by staining of active caspase-3. Quantification of B) apoptotic cells and C) necrotic cells on H&E stained tumor tissue sections using Definiens© TissueMap software.

Immunohistochemical detection of apoptotic cells in remaining tumor tissue via active caspase-3 as performed two weeks after the last treatment cycle showed high numbers of apoptotic cells in tumors from animals treated with ^213^Bi-anti-CD38-MAb but not in PBS controls (Fig. 7A/B). The same holds true for detection of necrotic cells in H&E stained tumor slices using TissueMap image analysis software (Fig. 7C). Furthermore, analysis of H&E stained slices of the major organs did not reveal any signs of toxicity (data not shown).

## DISCUSSION

Treatment of hematological malignancies such as leukemias, lymphomas or myeloma often involves application of monoclonal antibodies targeting cell surface antigens of malignant cell types. To improve efficacy of the anti-CD20-MAb (rituximab) and of the anti-CD33-MAb (HuM195, lintuzumab) used in treatment of non-Hodgkin's lymphoma (NHL) and acute myeloid leukemia (AML), respectively, coupling of radionuclides has turned out beneficial [21-24]. Moreover, radioimmunoconjugates targeting the cell surface antigens CD45 and CD66 of blood cells have successfully been used for conditioning in nonmyeloablative marrow transplantation [25, 26]. Because of good availability combined with long-term responses in a number of patients, β-emitters such as ^90^Y, ^131^I or ^188^Re are usually applied in radioimmunotherapeutic regimens. However, due to the relatively low linear energy transfer of β-particles, eradication of tumor cells often is inadequate resulting in frequent tumor relapse. Furthermore, crossfire irradiation originating from the comparatively long range of β-particles may damage neighbouring normal tissue causing dose-limiting myelosuppression and cardiopulmonary toxicities particularly at higher doses [27]. Therefore, high linear energy transfer (~100keV/μm) α-particle-emitting radionuclides increasingly arouse interest. Due to the short range (40-100 μm) of α-particles in tissue targeted therapy with α-emitters such as ^211^At, ^213^Bi and ^225^Ac is particularly promising in treatment of micrometastatic disease or eradication of single tumor cells [16, 17, 28]. To overcome shortcomings along with targeted β-emitter therapy, application of alpha-emitters is increasingly investigated also in therapy of hematological malignancies. Treatment of nude mice bearing human B-lymphoma xenografts with the α-emitter ^227^Th coupled to the anti-CD20 antibody rituximab was significantly more efficient than with the β-emitter ^90^Y (^90^Y-tiuxetan-ibritumomab) [29]. ^213^Bi-lintuzumab targeting CD33 has been successfully applied in clinical trials with patients suffering from acute myeloid leukemia [30, 31]. Moreover, radioimmunotherapy with the anti-CD45 antibody labelled with the α-emitters ^213^Bi or ^211^At for conditioning in non-myeloablative allogeneic marrow transplantation caused durable donor engraftment [32, 33].

Building on these encouraging results with α-emitters in treatment of hematological malignancies and due to the high expression of CD38 in malignant plasma cells we evaluated therapeutic efficacy of ^213^Bi-anti-CD38 radioimmunoconjugates in multiple myeloma both *in vitro* and *in vivo*. Treatment with ^213^Bi-anti-CD38-MAb induced cell death in myeloma cells dependent on the individual CD38 expression level in each cell line, i.e. effects were significantly higher in cells with high CD38 expression. Cell death is most likely caused by radiation-induced DNA double strand breaks. Exposure to ^213^Bi-radioimmunoconjugates induced cell cycle arrest in the G2-phase prior to cell death. As data on quantification of histone H3 phosphorylation using dual-parameter flow cytometry suggest, OPM2 cells are not strictly arrested in G2-phase after ^213^Bi-antiCD38-MAb treatment but enter or even complete M-phase (mitosis) before they die (Fig. 5A/B). Completion of mitosis with damaged DNA may be beneficial in case of generation of advantageous genomic rearrangements in surviving cells or of rearrangements that help cells to survive [34]. However, because the number of OPM2 cells in G2-phase constantly increased after ^213^Bi-anti-CD38-MAb treatment, the cells obviously did not generate beneficial genomic rearrangements and therefore they died after completion of mitosis at the latest. This again emphasizes the destructive potential of α-particle radiation towards tumor cells that can be successfully used for therapy. *In vivo*, treatment of nude mice bearing myeloma xenografts with ^213^Bi-antiCD38-MAb significantly prolonged survival compared to unspecific ^213^Bi-DTPA and mock-treatment with PBS (Fig. 6). One option to improve therapeutic efficacy might be to raise the number of treatment cycles. As we could demonstrate by histopathological examinations, major organs did not show any signs of toxicity even after six rounds of ^213^Bi-anti-CD38-MAb injection. Therefore, additional treatment cycles could possibly enhance the therapeutic success without affecting the normal organs. As we have shown previously, ^213^Bi-induced cell death does not occur before days 3 to 4 after treatment [35]. Thus, repetitive treatment cycles with ^213^Bi-imunoconjugates every third or fourth day could gradually diminish tumor size even of advanced tumors finally resulting in complete tumor eradication. Another option to improve therapeutic efficacy includes radioimmunotherapy using alpha-emitters with longer half-lives such as ^211^At (t_½_ = 7.2 h) and ^225^Ac (t_½_ = 10 d). Following i.v. injection of α-emitter immunoconjugates, as will be the method of application in patients suffering from MM, efficient targeting of malignant plasma cells usually requires time periods longer than one half-life of ^213^Bi (t_½_ = 46 min). Therefore, half-lives of α-emitters within the range of hours or even days coupled to anti-CD38 antibodies could ensure efficient irradiation of CD38-positive plasma cells resulting in cell death.

Nevertheless, intravenous injection of ^213^Bi-immunoconjugates targeting syndecan-1 (CD138), which is expressed at high levels in MM tumors within the bone marrow, was therapeutically effective in a murine MM model [36]. Finally, combination therapies of radioimmunoconjugates and cytostatic or molecular drugs are conceivable. Taken together our data suggest that α-emitter radioimmunotherapy is a promising future option for treatment of MM with tolerable side effects.

## MATERIALS AND METHODS

### Cell culture

The myeloma cell lines OPM2, ARH77, and RPMI8226 (all gift of T. Dechow, Technische Universität München) were cultured in RPMI 1640 medium (Biochrom, Berlin, Germany) supplemented with 10% fetal bovine serum, 100 U/ml penicillin, 100 μg/ml streptomycin and 1% L-glutamine (all from Biochrom). All cells were cultivated at 37°C in a humidified atmosphere with 5% CO_2_.

### Assessment of antibody-binding by flow cytometry

The QuantiBRITE^TM^ system was used to quantify the number of antibodies bound per cell (ABC). 1×10^5^ cells were incubated for 30 min at 4°C with QuantiBRITE™ anti-CD38 coupled with the red fluorescent protein phycoerythrin (PE) (labelling ratio: one molecule of PE / molecule of antibody). Flow cytometric analyses were performed on a FACSCalibur device using CellQuestPro software. Measured CD38 RFI-values were correlated to PE molecules using QuantiBRITE^TM^ PE-beads and conversion to the number of CD38 PE-ABCs was done with GraphPad PRISM^TM^ software.

### ^213^Bi labelling of anti-CD38 antibody

The ^213^Bi chelating agent SCN-CHX-A”-diethylenetriaminepentaacetic acid (DTPA) (Macrocyclics, USA) was covalently coupled to the anti-CD38-MAb MOR03087 (MorphoSys AG, Munich, Germany) as described previously, resulting in 3 - 5 molecules of chelating agent per antibody [37]. The α-emitter ^213^Bi was eluted from an ^225^Ac/^213^Bi generator system provided by the Institute for Transuranium Elements (European Commission, JRC, Germany) [38, 39]. Chelated anti-CD38-MAb (100 μg) was incubated with ^213^BiI_4_^−^/^213^BiI_5_^2-^ anionic species, as eluted from the generator, for 7 min in 0.4 M ammonium acetate buffer at pH 5.3. Unbound ^213^Bi ions were separated from ^213^Bi-anti-CD38-MAb immunoconjugates by size-exclusion chromatography (PD-10 columns, GE Healthcare, Germany). Purity of ^213^Bi-anti-CD38 immunoconjugates was tested by instant thin-layer chromatography [40] and was not less than 99%. Average specific activity attained was 740 kBq/μg of antibody. Binding of ^213^Bi-anti-CD38 immunoconjugates to multiple myeloma cells was analysed as described previously [41].

### Assessment of cell viability

OPM2, RPMI8226 and ARH77 cells (2×10^4^ in 100 μl culture medium per well) were seeded in 96-well plates and incubated with different ^213^Bi-anti-CD38-MAb activity concentrations ranging from from 46.3 kBq/ml (62.5 ng of antibody/ml) to 1.48 MBq/ml (2 μg of antibody/ml). At 48 h after initiation of incubation with ^213^Bi-anti-CD38-MAb at 37°C in a humidified atmosphere with 5% CO_2_, viability of cells was assayed in comparison to untreated cells using the CellTiter96® cell proliferation assay (Promega, Madison, USA). For that purpose 15 μl of dye solution was added to each well and the plates were incubated for 4 h at 37°C in a humidified CO_2_ incubator. Subsequently 100 μl of solubilisation/stop solution (formazan product) was added to each well and the absorbance was recorded one hour later at 570 nm using a 96-well plate reader (BioTek, Bad Friedrichshall, Germany) according to the manufacturer's instructions.

### Quantification of DNA double-strand breaks

OPM2 and ARH77 cells were seeded in 8-chamber slides covered with poly-L-lysin (2.5×10^4^ cells per chamber) and treated with ^213^Bi-anti-CD38-MAb (1.48 MBq/ml) for 3 h at 4°C to prevent DNA-repair. Cells were washed with PBS and incubated in culture medium at 37°C to allow DNA-repair. At indicated time points γH2AX was detected by immunofluorescence. For that purpose cells were fixed in 2% paraformaldehyde, washed with PBS and permeabilized with ice-cold methanol, washed with PBS and incubated with anti-γH2AX antibody (Millipore, Schwalbach/Ts, Germany; 1 h, RT) and anti-IgG antibody coupled with FITC (1 h, RT). Detection and quantification of immunofluorescence signals was done by image analysis using the Definiens Cognition Network Technology®.

### Flow cytometric analysis of cell-cycle arrest

OPM2 cells (5×10^6^ per 75 cm^2^ culture flask) were incubated with or without ^213^Bi-anti-CD38 immunoconjugates (1.48 MBq/ml) for 3 h at 37°C. Subsequently cells were washed once wit PBS and incubated in fresh culture medium for the indicated time periods. Cells were washed in PBS and fixed in 0.5 ml 80 % ethanol. For cell-cycle analysis fixed cells were incubated with RNase (0.1 mg/ml; 5 min RT), treated with pepsin (5 mg/ml in 50 mM HCl; 10 min 37°C), stained with PI (50 μg/ml) and subjected to flow cytometric analysis (FACScalibur, Becton Dickinson).

### Flow cytometric analysis of cell death

OPM2 cells (2×10^6^ per well of a 6-well plate) were incubated with or without ^213^Bi-anti-CD38 immunoconjugates (0.74 MBq/ml). At 120 h after treatment cells were washed once with PBS and fixed in ice-cold 70% ethanol. Cells were washed again with PBS and incubated with anti-phospho-histone-H3 antibody (1:200; Cell Signaling Technology / New England Biolabs, Frankfurt, Germany) in 1% BSA for 3 h at room temperature (RT). After washing with PBS cells were incubated with the secondary anti-IgG antibody coupled with FITC (from rabbit, 1:1000; Abcam, Cambridge, UK) for 1 h at RT. Finally cells were washed with PBS and resuspended in 5 μg/ml propidium iodide (PI) + RNase 0.1%. PI and FITC fluorescence of cells were analyzed by dual-parameter flow cytometry.

### Western blotting

OPM2 cells (2×10^6^ per well of a 6-well plate) were incubated with ^213^Bi-anti-CD38-MAb (0.74 MBq/ml). At different time points after start of incubation, i.e. at 0, 5, 9, 24, 48, 72, 96 and 120 h, cells were washed in PBS and subsequently lysed (50 mM Tris, pH 7.5; 250 mM NaCl; 0.1% Triton X-100; 1 mM EDTA; 50 mM NaF + protease inhibitors) at 4°C for 10 min. Lysates were centrifuged (13,500 rpm, 4°C, 10 min) and supernatants (containing 25 μg of protein each; BCA protein assay kit, Pierce, USA) were subjected to SDS-PAGE. Western blotting using different antibodies against clapsin (gift from Michele Pagano), cdc20 (Santa Cruz Biotechnology, Heidelberg, Germany), p27 (BD Biosciences, Heidelberg, Germany), Plk1 (Invitrogen / Life Technologies, Darmstadt, Germany), wee1, cyclin B1, p-HH3, BimEL, Bax, cleaved PARP (all from Cell Signaling Technology / New England Biolabs, Frankfurt, Germany), γH2AX, active caspase-3 (all from Millipore, Schwalbach/Ts, Germany), peroxidase-conjugated monoclonal anti-β-actin antibody (clone 8226, Abcam, Cambridge, UK) and peroxidase-conjugated anti-rabbit IgG antibody (GE Healthcare, Hatfield, UK) was performed as described previously [42].

### Preclincal treatment study

OPM2-Luc-GFP cells (2.5×10^7^ in 100 μl PBS), as generated via lentiviral transduction [43], were inoculated intraperitoneally into 6-8 week-old SCID-mice (Charles River, Germany). Between days 25 and 42 after cell inoculation tumor bearing animals showing several tumor centres due to i.p. inoculation (n = 9) received six intraperitoneal applications of ^213^Bi-anti-CD38-MAb (1.85 MBq each in 100 μl PBS) every third or fourth day. For control of antibody specificity, another group of animals (n = 6) received six injections of ^213^Bi-DTPA (1.85 MBq each in 100 μl PBS) not targeting OPM2 cells. Control animals (n = 9) were injected six times with 100 μl PBS. Efficacy of therapy was controlled non-invasively in two mice each of the treatment group and the control group by bioluminescence imaging of tumor development 34, 47, and 59 days after tumor cell inoculation. Thus, bioluminescence imaging was performed two days after the third treatment (d 34) and five as well as 17 days after the sixth treatment (d 47, d 59). Imaging was done with anesthetized mice 10 min after intraperitoneal injection of 300 μl D-luciferin (50 mM in 0.9% NaCl) using a cooled CCD-camera (Hamamatsu, Germany). Symptom-free survival was monitored up to 200 days after inoculation of tumor cells.

### Histopathologic assessment of tumor cell death

Two weeks after the final treatment cycle, two mice each of the treatment group (^213^Bi-anti-CD38-MAb) and the control group (PBS) were sacrificed and the remaining tumor tissue as well as different organs (heart, liver, lung, spleen, pancreas, bone, brain, kidneys, stomach and intestine) were dissected, weighed and fixed in 4% buffered formalin. Organs and tumors were embedded in paraffin. Slices of the major organs (4 μm) were stained with haematoxylin and eosin (H&E) and subjected to toxicity analysis. For detection and quantification of necrosis 1 μm thick paraffin slices were stained with H&E and evaluated with TissueMap image analysis software (Definiens®, Munich, Germany). For immunohistochemical detection of apoptotic cells, paraffin slices were dewaxed, rehydrated and incubated with an anti-caspase-3 antibody (1:100; Abcam, Cambridge, UK) for 2 h at room temperature. Anti-caspase-3 antibody binding was verified using a secondary antibody labelled with horseradish peroxidase and DAB (3,3-diaminobenzidine tetrahydrochloride) as a substrate (DAB Detection Kit, Roche-Ventana, Penzberg, Germany). Immunohistochemical analysis was performed automatically using the CC1 program of the immunostainer Discovery XT device (Roche-Ventana, Penzberg, Germany). Images were acquired using a virtual microscope system (Olympus-Dotslide, version 2.0, Hamburg, Germany). Finally, quantification of the percentages of apoptotic cells present in the tumor sections was done using Definiens TissueMap® software.

### Statistical analysis

Software package SPSS Statistics version 17.0.0 for Microsoft Windows XP (SP3) was used for statistical analysis. Significance of survival as expressed in Kaplan-Meier curves was determined using the log-rank test. The level of statistical significance was set at p < 0.05.
